# Personal exposure measurements of school-children to fine particulate matter (PM_2.5_) in winter of 2013, Shanghai, China

**DOI:** 10.1371/journal.pone.0193586

**Published:** 2018-04-02

**Authors:** Lijun Zhang, Changyi Guo, Xiaodong Jia, Huihui Xu, Meizhu Pan, Dong Xu, Xianbiao Shen, Jianghua Zhang, Jianguo Tan, Hailei Qian, Chunyang Dong, Yewen Shi, Xiaodan Zhou, Chen Wu

**Affiliations:** 1 Environmental Health Department, Division of Heath Risk Factors Monitoring and Control, Shanghai Municipal Center for Disease Control and Prevention/Shanghai Institutes of Preventive Medicine, Shanghai, China; 2 General Office, Shanghai Municipal Center for Disease Control and Prevention/Shanghai Institute for Prevention Medicine, Shanghai, China; 3 Division of Heath Risk Factors Monitoring and Control, Shanghai Municipal Center for Disease Control and Prevention/Shanghai Institutes of Preventive Medicine, Shanghai, China; 4 Environmental Health Department, Shanghai Xuhui Center for Disease Control and Prevention, Shanghai, China; 5 Environmental Health Department, Shanghai Baoshan Center for Disease Control and Prevention, Shanghai, China; 6 Shanghai Key Laboratory of Meteorology and Health, Shanghai Meteorological Service, Shanghai, China; The Ohio State University, UNITED STATES

## Abstract

**Objective:**

The aim of this study was to perform an exposure assessment of PM_2.5_ (particulate matter less than 2.5μm in aerodynamic diameter) among children and to explore the potential sources of exposure from both indoor and outdoor environments.

**Methods:**

In terms of real-time exposure measurements of PM_2.5_, we collected data from 57 children aged 8–12 years (9.64 ± 0.93 years) in two schools in Shanghai, China. Simultaneously, questionnaire surveys and time-activity diaries were used to estimate the environment at home and daily time-activity patterns in order to estimate the exposure dose of PM_2.5_ in these children_._ Principle component regression analysis was used to explore the influence of potential sources of PM_2.5_ exposure.

**Results:**

All the median personal exposure and microenvironment PM_2.5_ concentrations greatly exceeded the daily 24-h PM_2.5_ Ambient Air Quality Standards of China, the USA, and the World Health Organization (WHO). The median *E*_*total*_ (the sum of the PM_2.5_ exposure levels in different microenvironment and fractional time) of all students was 3014.13 (μg.h)/m^3^. The concentration of time-weighted average (*TWA*) exposure of all students was 137.01 μg/m^3^. The median *TWA* exposure level during the on-campus period (135.81 μg/m^3^) was significantly higher than the off-campus period (115.50 μg/m^3^, *P* = 0.013 < 0.05). Besides ambient air pollution and meteorological conditions, storey height of the classroom and mode of transportation to school were significantly correlated with children’s daily PM_2.5_ exposure.

**Conclusions:**

Children in the two selected schools were exposed to high concentrations of PM_2.5_ in winter of 2013 in Shanghai. Their personal PM_2.5_ exposure was mainly associated with ambient air conditions, storey height of the classroom, and children’s transportation mode to school.

## Introduction

Numerous epidemiological studies have shown that long-term or short-term exposure to PM_2.5_ can result in a wide spectrum of adverse health effects, such as respiratory diseases, cardiovascular disease and excess mortality[1–5]. When breathed, PM_2.5_ can reach the bloodstream and translocate to vital organs such as the liver, spleen, heart and so on. It can lead to diverse adverse health outcomes include impaired pulmonary function, increased blood pressure, stroke, lung cancer, and some other illnesses. Scientists suggest that PM_2.5_ is more harmful to human health than other coarse particles [6].

With the development of rapid urbanization and industrialization, China experienced a dramatic increase in energy consumption and emission over the past few decades. And at the same time, ambient PM_2.5_ air pollution has become one of the environmental challenges in recent several years[7]. According to the global estimate of ambient PM_2.5_ concentrations from satellite-based aerosol optical depth, the level of PM_2.5_ in China represents one of the highest in the world [8].The Global Burden of Disease Study showed that ambient PM pollution was the fourth leading risk factor for disability-adjusted life-years (DALYs) in China in 2010, which resulted in 25.2 million DALYs [9].

Although Shanghai is the most economically developed and rapidly urbanizing city in eastern China, air pollution was severe in recent years due to the high levels of energy consumption, large emissions of atmospheric pollutants, and the influence of the East Asian monsoon on long-range transport in the Yangtze River Delta(YRD) region[10, 11]. Furthermore, an unusual air pollution episode occurred in Shanghai in winter of 2013. Air quality limits were frequently exceeded and exceptionally high particulate matter mass concentrations were frequently recorded. According to the report of Ming L *et al*. [10], three heavy pollution events were occurred from 5 November in 2013 to 7 January in 2014 in Shanghai and PM_2.5_ concentrations even peaked at an extremely high level of 395μg/m^3^ on 6 December in 2013.

Children are known to be vulnerable to contaminated air during activities in different micro-environments [12]. Therefore, children’s exposure to environmental contaminants is often very different in many instances, and tends to be much higher than in adults [13–15]. For that reason, it is generally agreed that exposure assessment of PM_2.5_ in school-children is much more complex and challenging. However, school-children have relatively fixed time-activity patterns within a certain micro-environment through on-campus sites (e.g. classroom, playground, and corridors) or off-campus sites (e.g. on pavements to school, indoors and outdoors at home). Moreover, micro-activities performed by school-children are relatively limited, and include learning, eating, sleeping, playing games, and watching television. Therefore, it is highly desirable to evaluate exposure levels among children using an indirect exposure assessment. This indirect exposure assessment method has been reported more than twenty years ago and has been used for measurement of many air pollutants such as benzene exposure, carbon monoxide (CO) exposure, and particle exposure [16, 17], which used validated microenvironment models and human activity pattern data obtained from questionnaires to predict exposure levels in a certain population [18].

Given that there were few studies which have carefully looked at the relationship between personal activity and exposure levels to PM_2.5_ with respect to time and real-time pollutant monitoring, especially in China, we conducted personal exposure level assessments of PM_2.5_ among school-children in two schools in Shanghai. Particularly, this research was conducted in the episode of 2013 exactly and this article intends to highlight the personal exposure level of PM_2.5_ among school students in this period by means of micro-environmental monitoring and personal sampling, and to explore influence of potential sources of PM_2.5_ exposure in both indoor and outdoor environments.

## Materials and methods

### Study participants and design

This study was designed to continuously monitor changes in the PM_2.5_ level, while simultaneously recording activities among school-children at school and home. Based on air pollution and human health monitoring area data in Shanghai and the cluster sampling principle, two primary schools were selected. School A is located in the downtown Shanghai and situated 1 km west of the monitoring spot at the Shanghai Normal University, Xuhui district, while School B is located in the suburban area of Baoshan district and situated 4.7 km north of the monitoring spot at Hongkong Liangcheng area. Following the principles of gender equity and voluntary participation, 57 children of the 3^rd^, 4^th^ and 5^th^ grade from the two primary schools were recruited. All students lived in two communities close to the two schools. Our study was approved by the ethics committee of Shanghai Municipal Centre for Disease Control and Prevention (IORG No: IORG0000630). Written informed consent was obtained from the parent or guardian of each participant before the study was initiated. The protocol included four parts.

#### Questionnaire survey

We conducted a cross-sectional survey on indoor and outdoor environment among these children. The main variables included general information, living condition, and lifestyle information.

#### PM_2.5_ measurements inside the campus microenvironment

Continuous PM_2.5_ measurements were collected simultaneously from several micro-environments inside the school where students stayed (e.g. classrooms, main corridors, and the playgrounds). TSI DUSTTRAK^TM^ DRX (Model 8533, TSI Inc. Paul, MN, USA) apparatus was set up in each environment from about 8 a.m. to 4 p.m. (Investigations in Schools A and B were taken between November 20th to 28th and from December 18th to 26th in 2013, respectively) and calibrated with the manufacturer’s high-efficiency particulate filter before sampling. One sampler was placed in the playground and the other was placed in the main corridor, respectively for 6 days during the working days. Two samplers were placed in each classroom for continuous two days based on the diagonal distribution principle, with one in the front of the classroom and another in the rear. All sampling instruments were placed approximately 1 meter away from walls and other barriers, and were at a height of 1.2 meters above ground level, which was approximately at the height of the breathing zone of children. The sampling flow rate was 3 l/min, and values were recorded continuously over 8 hours with time intervals of 1 min. Recorded values were corrected to 1 μg/m^3^, and the limit of detection (LOD) was 1μg/m^3^. During the sampling period, all classrooms retained ventilation habits as usual, including the opening and closing of windows and doors.

#### Personal measurements outside campus

During the off-campus period (from 4 p.m. to 6 a.m. the next day), PM_2.5_ concentrations were measured by a set of real-time laser diode photometers (model no. SidePak^TM^ AM510, TSI Inc, USA), which were placed in small bags. A sampling air inlet was fixed in the vicinity of the students’ breath zone. All participants were asked to carry the sampling bags from about 4 p.m. to 8 a.m. the next day, although this excluded the sleeping and showering periods. When the monitor was taken off, it was placed within the breathing zone of the subjects. All students returned sampling bags on the next morning. Each instrument was cleaned, greased and batteries were replaced after daily sampling. All instruments were reset daily before sampling, according to the manufacturer’s instructions. After school the next day, the same sampling bags were re-distributed to the same students who took the sampling bags on the first day. All participating students were required to finish personal sampling for a continuous 2 days period. Meanwhile, ambient air pollutants concentration (e.g. PM_10_, PM_2.5_, NO_2_ and SO_2_) and meteorological indicators (e.g. temperature, humidity, and wind speed) in the two monitoring spots closed to the schools were collected from the Real-time Air Quality Reporting System of Shanghai Environmental Monitoring Centre (http://www.semc.com.cn/aqi/home/Index.aspx) and Shanghai Meteorological Service, respectively.

#### Time-activity diary investigation

Each student was asked to finish a diary questionnaire that included time, activities, and location. The on-campus part of the time-activity questionnaire was based on the school timetable, and the off-campus part was recorded every 30 minutes according to individual schedules by participants.

### Statistical analysis

Real-time PM_2.5_ concentrations were calculated by taking the average PM_2.5_ mass concentration obtained from the gravimetric method for the sake of reliability. Gravimetric PM_2.5_ was measured according to the method stipulated by the Environmental Protection Agency of United State (U.S. EPA) and research conducted by Jiang et.al. [19]. In this investigation, linear regression analysis indicated that the PM_2.5_ concentration could be measured by two laser diode photometer measurements with linear regression models of y = 0.602x+0.774 (n = 21, *R*^*2*^ = 0.799, *P* <0.001) and y = 0.577x-1.467 (n = 21, *R*^*2*^ = 0.769, *P* <0.001). On average, the PM_2.5_ concentrations reported by the two laser diode photometer monitors in our research were approximately 2.25- and 2.59- fold as high as the gravimetric measurements (Please refer to Supporting information file S1 Protocol).

The exposure level (*E*_*total*_) was calculated as the sum of the partial exposure levels according to the relationship described by Eq 1 [18](where *Ci* is the concentration in the *i*th microenvironments, *Ti* is the fractional time spent in the *i*th microenvironment, and *N* is the number of microenvironments.)

$$E_{total}=\sum_{i}^{n}C_{i}T_{i}$$ (1)

*TWA* is the time-weighted average exposure, which means the average concentrations weighted on the integration period described by Eq 2.

$$TWA=E_{total}/T_{total}TWA$$ (2)

Principal regression analysis was used to explore indoor and outdoor impact of ambient PM_2.5_ pollution, meteorology and individual activities on personal PM_2.5_ exposure levels. Students’ PM_2.5_ total exposure concentrations (*E*_*total*_) over the two days were treated as the dependent variables, while other potential factors were treated as the independent variables. The latter included ambient air pollutant concentrations, meteorological factors, school position, sex, age, height, weight, family member status, mode of transportation to school, exercise, second-hand smoke exposure, vehicles near houses and other variables.

All data were examined for validity and complied with our standard operating procedures. Flagged data were removed when battery failure or disconnected power supply was detected. All statistical analysis was performed by SAS for Windows (version 9.4; SAS Institute Inc., 2003) and the level of significance was defined as *P* <0.05 (2-tailed).

## Results

### Basic participant characteristics

57 students participated in the investigation with 30 (52.6%) children in school A and 27 (47.4%) children in school B. Table 1 summarized basic characteristics of the students and their indoor and outdoor environmental conditions. There were 22 (38.6%) males and 35 (61.4%) females. The average age, height and weight of the children was 9.64 ± 0.93 years old, 140.77 ± 8.53 cm and 37.81 ± 9.69 kg, respectively. Among these, only 56 children finished the questionnaire survey. The questionnaire results showed that 36 (64.3%) children comprised families of three people. Among the investigated students, per capita living space of 43(75.4%) students was over 30 square meters. Most of the children (71.4%) lived between floors 3 to 10. 25 (44.6%) children had exposure to second-hand smoke in the family home. 40 (70.2%) children lived by the side of heavy-traffic roads and 14 (24.6%) children lived near large factories. In spring and winter, 51 (91.1%) homes tended to report opening the window(s) every day. 47 (83.9%) children often exercised. 48 (85.7%) children were from families that used smoke exhaust ventilator in their kitchen every day. Children’s mode of transportation to school varied, which was 24 (42.9%) on foot i.e. walking, 20 (35.7%) by bike and 12 (21.4%) by bus, car or subway.

**Table 1:** Basic characteristics of the students.

Variable	Values
School	
A	30(52.6)
B	27(47.4)
Gender	
male	22(38.6)
female	35(61.4)
Age (years)	
mean	9.64±0.93
range	8.08–12.00
Height (cm)	
mean	140.77±8.53
range	124.00–159.00
Weight (kg)	
mean	37.81±9.69
range	25.00–68.00
Family members^a^	
three	36(64.3)
four	7(12.5)
five+	13(23.2)
Per capita living space^a^	
< = 30 square meters	13(22.8)
>30 square meters	43(75.4)
Living floor^a^	
Above 10th Floor	7(12.5)
3rd-10th Floor	40(71.4)
Below 3rd floor	9(16.1)
Second-hand smoke^a^	
yes	25(44.6)
no	31(55.4)
Neighborhood road vehicle numbers^a^	
more	40(70.2)
less	16(28.1)
Large factory in neighborhood ^a^	
Yes	14(24.6)
No	42(73.7)
Frequency of opening of window(s) in spring and winter^a^	
every day	51(91.1)
Once every 2–3 days	5(8.9)
The use of exhaust smoke system in kitchen^a^	
Every day	48(85.7)
occasionally	3(5.4)
never	5(8.9)
Exercise^a^	
often	47(83.9)
hardly	9(16.1)
Mode of transportation to school^a^	
walking	24(42.9)
riding a bicycle	20(35.7)
taking bus/car/subway	12(21.4)

[Note]

^a^ 56 children completed the questionnaire investigation.

### Micro-environmental and personal exposure

Table 2 summarizes the real-time concentration (calibrated results) of PM_2.5_ in different microenvironments in the two schools. Median and inter-quartile ranges (*P*_*25*_*-P*_*75*_) were reported together with the arithmetic means and these were used to describe the distribution due to the right-skewed distribution of all measurements. All the median personal exposure and microenvironment PM_2.5_ concentrations greatly exceeded the secondary standard of daily 24-h PM_2.5_ Ambient Air Quality Standards of China (75μg/ m^3^)[20], the current U.S. EPA daily ambient standard of 35 μg/m^3^ [21], and the WHO Air Quality Guideline of 25μg/m^3^[22]. The median PM_2.5_ level in the rear classroom microenvironments in school A was 139.84 μg/m^3^. In comparison, the median PM_2.5_ level in school B was 144.65 μg/m^3^, which was significantly higher than in school A. However, the PM_2.5_ levels in other microenvironments in school B were not significantly different than in school A. Moreover, school B seemed to have an extremely high concentration of PM_2.5_ at the personal exposure level, which was 131.65 μg/m^3^, while the exposure level in school A was much lower, with a median value of 103.25 μg/m^3^ (*P* < 0.001).

**Table 2:** Concentration of PM_2.5_ exposure obtained from exposure measurements (μg/m^3^).

Type	School	Spot	N^a^	Mean	SD	Median	*P*_*25*_*—P*_*75*_
Micro-environment measurements	A	classroom_front	2880	163.16	71.40	148.26	119.37–207.26
		classroom_rear	2858	160.35	83.74	139.84	94.69–211.47
		corridor	2148	131.91	53.70	116.96	101.91–171.14
		playground	2878	155.37	75.70	129.60	106.73–202.44
	B	classroom_front	2876	220.41	192.60	147.06	97.70–210.87
		classroom_rear	2834	218.78	185.36	144.65**	100.71–215.69
		corridor	2880	186.49	170.87	117.56	84.45–172.95
		playground	2823	202.76	182.95	130.20	94.08–191.01
Personal exposure measurements	A	personal	40268	137.98	107.17	103.25	66.49–174.55
	B	personal	37387	159.18	112.88	131.65**	90.44–200.72

Note

^a^ Exposure measurement values of a few time points were not available due to instrument battery failure.

**Compared with School A and analyzed using Wilcoxon test, *P* <0.001.

Table 3 summarizes the ambient fixed monitoring data obtained from Shanghai Environmental Monitoring Centre and Shanghai Meteorological Service for the same period of exposure measurements. Ambient PM_2.5_ and SO_2_ concentrations in the fixed monitoring spot and some meteorological indicators such as relative humidity (RH) and wind speed (WS) close to school B were significantly higher than for school A (*P* < 0.001), while temperatures close to school B were significantly lower than for school A (*P* < 0.001).

**Table 3:** Concentration of ambient air pollutants and meteorological data obtained from fixed monitoring spots.

Type	School	Spot	N	Mean	SD	Median	*P*_*25—*_*P*_*75*_
Air pollutant monitoring data (μg/m^3^)	A	PM_2.5_	136	98.01	41.64	90.80	66.58–122.53
		PM_10_	136	188.54	98.80	149.75	109.30–255.73
		SO_2_	133	43.13	18.96	40.25	31.93–48.63
		NO_2_	132	81.38	32.70	78.65	53.40–101.28
	B	PM_2.5_	140	138.66	86.01	112.25 **	76.70–193.13
		PM_10_	137	170.27	96.01	140.80	96.03–218.80
		SO_2_	136	60.73	32.18	47.55 **	36.63–77.40
		NO_2_	137	84.26	24.35	89.10	61.63–100.25
Meteorological monitoring data	A	Temperature(°C)	144	10.72	3.99	11.30	7.60–13.60
		RH(%)	144	44.71	16.06	40.00	33.00–57.00
		WS(m/s)	144	0.94	0.66	0.90	0.50–1.30
	B	Temperature(°C)	144	4.53	2.44	4.60**	2.73–6.20
		RH(%)	144	64.44	10.23	65.00**	58.00–72.00
		WS(m/s)	144	4.08	2.20	4.00 **	2.40–5.80

Note

**Compared with School A and analyzed using Wilcoxon test, *P* <0.001.

### Time-activity pattern survey

During the on-campus period, the constituent ratio of time-activity patterns in the different micro-environments in the two schools were significantly different (χ^2^ = 26.988, *P* <0.001). Students in school A spent much more time in classroom, which concomitantly resulted in less time in the playground, corridors and other places compared with those students in school B. In particular, students in school A spent duration of 377 mins in the classroom compared with students in school B who spent 355 mins in classroom. We calculated a constituent ratio for the average time spent in different micro-environments such as the bedroom, dining room, bath room, kitchen, being on the road and other places. Students in school A spent 960 mins during the off-campus period in particular micro-environments, which was 706 mins (73.5%) in the bedroom, 113 mins (11.8%) in the dining room, 36 mins (0.3%) in the bath room, 3 mins (0.3%) in the kitchen, 51 mins (5.3%) on the road, and 51 mins (5.3%) in other places, respectively. Students in school B spent 930 mins during the off-campus period in the following micro-environments: 701 mins (75.3%) in the bedroom, 103 mins (11.1%) in the dining room, 35 mins (3.8%) in the bath room, 9 mins (1.0%) in the kitchen, 33 mins (3.5%) on the road, and 50 mins (5.4%) in other places, respectively. The constituent ratio for the time-activity patterns in different micro-environments during the off-campus periods between two school students were not significantly different (χ^2^ = 6.919, *P* = 0.227).

### Variations in the average 24-hour PM_2.5_ concentrations, ambient pollution and meteorological indicators

**Fig 1:**
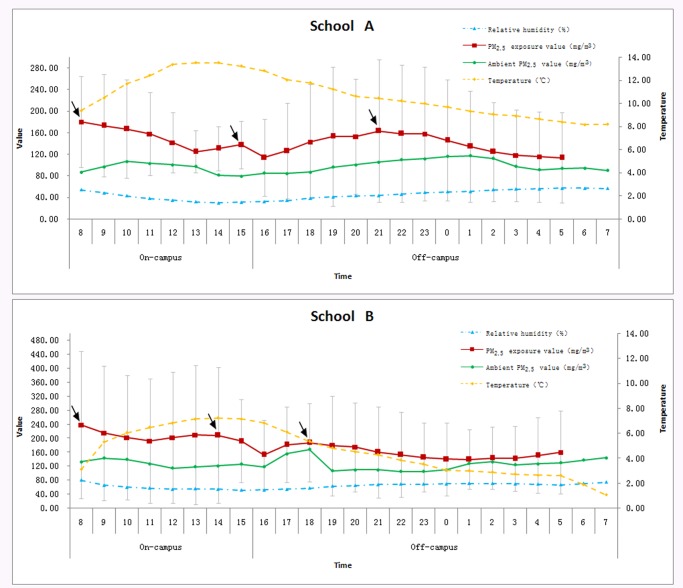
Variations in the average 24-hour PM_2.5_ concentrations, ambient pollution and meteorological indicators.

Fig 1 shows the personal PM_2.5_ exposure concentrations and hourly PM_2.5_ data from continuous monitors, which were averaged over a 22-hr period to match the outside fixed monitoring data and meteorology indicators (temperature and relative humidity).

PM_2.5_ exposure concentrations during the on-campus period were all much higher than ambient PM_2.5_ concentrations in closed environment monitoring spots during the investigation period. Two peaks were detected at 8 a.m. and 3 p.m. or 2 p.m. during the on-campus period. During the off-campus period, the variations in personal exposure of students to PM_2.5_ between the two schools were different. Students in school A seemed to have a similar exposure level to that of ambient PM_2.5_ exposure. Furthermore, students’ personal exposure level peaked at 9 p.m. and started to decline from 9 p.m. to 5 a.m. the next day. Students’ personal exposure level in school B peaked at 6 p.m. and started to decline from 6 p.m. to 1 a.m. the next day.

### PM_2.5_ exposure level of school students

Based on micro-environmental PM_2.5_ monitoring results, personal sampling and students’ time-activity patterns, the PM_2.5_ exposure levels for all students during the on-campus and off-campus school periods were calculated (Table 4). The median *E*_*total*_ of all students in a 22-hr period was 3014.13 (μg.h)/m^3^. The median *E*_*total*_ of school A and school B were 2783.77 (μg.h)/m^3^ and 3404.60 (μg.h)/m^3^ respectively. The median *TWA* in a 22-hr period of all students was 137.01 μg/m^3^. The median *TWA* of school A and school B were 126.53 μg/m^3^ and 154.75 μg/m^3^ respectively. No significant differences in the median *E*_*total*_ and *TWA* in a 22-hr period were observed between the two schools. During the on-campus period, the median *TWA* of all students was 135.81 μg/m^3^, which was significantly higher than the off-campus period value (115.50 μg/m^3^, *P* = 0.013 < 0.05). As for school A, the median *TWA* during the on-campus was also significantly higher than the off-campus period value (*P* = 0.017 < 0.05). No significant differences in median *TWA* were observed between the two schools.

**Table 4:** Exposure levels of PM_2.5_ among the children.

School	Statistics	On-campus Period		Off-campus Period^a^		Total^a^	
		*E*_*total*_	*TWA*	*E*_*total*_	*TWA*	*E*_*total*_	*TWA*
		[(μg.h)/m^3^]	(μg/m^3^)	[(μg.h)/m^3^]	(μg/m^3^)	[(μg.h)/m^3^]	(μg/m^3^)
A	N	60	60	52	52	52	52
	Mean	1210.78	151.35	1879.85	134.27	3132.61	142.39
	SD.	453.43	56.68	1209.29	86.38	1505.98	68.45
	Median	1148.19	143.52	1502.02	107.29*	2783.77	126.53
	*P*_*25*_	972.42	121.55	1010.75	72.20	1991.72	90.53
	*P*_*75*_	1461.55	182.69	2616.16	186.87	3889.11	176.78
B	N	54	54	51	51	51	51
	Mean	1807.10	212.60	1974.71	146.27	3725.85	169.36
	SD.	1608.29	189.21	1099.30	81.43	1651.93	75.09
	Median	1154.38	135.81	1638.25	121.35	3404.60	154.75
	*P*_*25*_	778.76	91.62	1296.11	96.01	2327.90	105.81
	*P*_*75*_	1740.95	204.82	2698.49	199.89	4760.57	216.39
Total	N	114	114	103	103	103	103
	Mean	1493.25	180.36	1926.82	140.22	3426.35	155.74
	SD.	1187.42	139.33	1151.45	83.77	1600.16	72.73
	Median	1154.38	135.81	1570.57	115.50^#^	3014.13	137.01
	*P*_*25*_	920.92	113.66	1098.48	78.74	2165.98	98.45
	*P*_*75*_	1531.83	188.32	2693.17	195.15	4399.46	199.98

[note]

^a^ Exposure measurement values of a few children were not available due to instrument battery failure.

*Compared with on-campus period, analyzed by Wilcoxon test, *P* = 0.017<0.05.

^#^Compared with on-campus period, analyzed by Wilcoxon test, *P* = 0.013<0.05.

### Correlation between ambient air pollutants, meteorological data and exposure levels

Spearman correlation coefficients were used to compare ambient air pollutants, meteorological data and exposure levels (Table 5). The high correlations were observed between on-campus PM_2.5_ exposure levels (*E*_*total*__on-campus) and ambient PM_2.5_ (*r* = 0.769), SO_2_ (*r* = 0.709), NO_2_ (*r* = 0.306), and PM_10_(*r* = 0.281). Similarly, the off-campus PM_2.5_ exposure level (*E*_*total*__ off-campus) correlated with ambient air PM_2.5_ (*r* = 0.361) and NO_2_ (*r* = 0.543). Moreover, a strong correlation between air pollutants and meteorological indicators were observed. Hence, there was strong co-linearity between exposure levels and ambient air pollutant concentrations, and meteorological indicators.

**Table 5:** Correlation between ambient air pollutants, meteorological data and exposure levels.

Variables	PM_2.5_	PM_10_	SO_2_	NO_2_	Temperature	RH	WS	*E*_*total*__on-campus	*E*_*total*__off-campus
PM_2.5_	1.000	0.465**	0.767**	0.708**	-0.339	0.210*	0.271**	0.769**	0.361**
PM_10_		1.000	0.453**	0.296**	-0.264	-0.643	-0.124	0.281**	0.115
SO_2_			1.000	0.459**	-0.288	-0.110	0.140	0.709**	0.130
NO_2_				1.000	0.265**	0.012	-0.312	0.306**	0.543**
Temperature					1.000	-0.288	-0.729	-0.240	0.047
RH						1.000	0.624**	0.225*	0.096
WS							1.000	0.464**	-0.151
*E*_*total*__on-campus								1.000	-0.055
*E*_*total*__off-campus									1.000

Note: Analyzed using Spearman rank test

*P <0.05

**P <0.01

### Principal component analysis (PCA)

PCA was used to eliminate co-linearity between exposure levels and ambient air pollutant concentrations, and monitored meteorological indicators [23]. We estimated a correlation matrix incorporating a total of 7 variables including PM_2.5_, PM_10_, SO_2_, NO_2,_ temperature, RH, and WS. Two PCs were extracted to interpret the original datasets after the varimax rotation. The two PC_S_ could explain approximately 72% of the total variances noted for the original data concourses. PC1 mainly reflected the information that was associated with ambient air pollution (i.e. PM_2.5_, PM_10_, SO_2_, and NO_2_). PC2 reflected the information related to meteorological conditions (i.e. temperature, RH, and WS).

### Multiple linear regression

We explored influence factors of children’s PM_2.5_ total exposure by multiple linear regression (Table 6). Children’s personal PM_2.5_ exposure in the two schools was associated with various factors. During the on-campus period, ambient air pollution (PC1) and meteorological conditions (PC2) were strongly correlated with *E*_*total*_ (*P <*0.0001). Storey height of the classroom was negatively correlated with students’ on-campus personal PM_2.5_ *E*_*total*_ levels (*P* = 0.0316). The regression equation was statistically significant (adjust *R*^*2*^ = 0.585 *F* = 50.33, *P* < 0.001). During the off-campus period, ambient air pollution (PC1) and children’s age were all positively correlated with students’ *E*_*total*_ exposure level (*P* = 0.0027 and *P* = 0.0260). However, students’ mode of transportation to school was negatively correlated with students’ *E*_*total*_ level (*P* = 0.0266). The regression equation was statistically significant (adjusted *R*^*2*^ = 0.168, *F* = 6.46, *P* = 0.0005).

**Table 6:** Impact exploring PM_2.5_ exposure by multiple linear regression.

		Unstandardized Coefficients						
Categoried	Variables	B	Std	t	*P*	*95% CI*	*F*	*R*^*2*^
On-campus	PC1	495.65	45.98	10.78	< .0001	404.50–586.80	50.33	0.585
	PC2	254.60	58.98	4.32	< .0001	137.67–371.53		
	Storey height of classroom	-206.72	94.90	-2.18	0.0316	-394.86 - -18.59		
Off-campus	PC1	197.93	64.34	3.08	0.0027	70.22–325.64	6.46	0.168
	Age	270.75	119.74	2.26	0.0260	33.08–508.43		
	Transportation mode to school	-308.75	137.09	-2.25	0.0266	-580.87 - -36.64		

## Discussion

The importance of indoor air quality (IAQ) in school environments has been globally highlighted [24, 25]. In our investigation, the PM_2.5_ concentration of all school micro-environments greatly exceeded the secondary standard of daily 24-h PM_2.5_ Ambient Air Quality Standards of China (75μg/ m^3^)[20], the current U.S. EPA daily ambient standard of 35 μg/m^3^ [21], and the WHO Air Quality Guideline of 25μg/m^3^[22]. Moreover, the average PM_2.5_ concentration in the classrooms was higher than in the corridors and the playgrounds. Furthermore, it was obvious that the average PM_2.5_ concentration was higher in the front of classrooms than in the rear of classrooms, which was probably caused by proximity to the blackboard position and more exhaled aerosols from teachers and students in this direction. Moreover, the corridor was a relatively semi-closed micro-environment, which was partly exposed to external ambient environment but without intensive or long-term human activities. Therefore, PM_2.5_ concentrations in the corridors are lower than those in the classrooms. The playgrounds were open-air microenvironments, and the PM_2.5_ concentration was equivalent to ambient PM_2.5_ levels except for those during centralized physical activities. These included the morning exercise period and physical activity times. Rovelli *et al*. claimed that one possible reason for PM_2.5_ pollution in the classroom was the re-suspension of settled particles due to insufficient ventilation, frequently cleaned indoor surfaces, and the presence of children and their movement [25]. Fromme *et al*. also found a higher average classroom PM_2.5_ concentration compared with the corresponding outdoor level at a German primary school [26]. Therefore, classroom air quality is one of the key aspects in exposure analysis and assessment of students’ PM_2.5_ exposure, air pollution control and health precautions.

As reported by de Oliveira *et al*., children between the ages of 6 and 14 were exposed to a higher PM_2.5_ dose during the dry season than during the rainy season [27]. Winter in Shanghai is generally considered to be the dry haze season because there is less rain and relative humidity was <80% often[28]. In our study, children’s daily PM_2.5_ exposure level reached a median value of 137.01 μg/m^3^. According to monitoring data from the Shanghai EPA, air quality during December in 2013 in Shanghai was rated ‘extremely serious’, which may consequently lead to higher PM_2.5_ exposure in primary school students. According to Brown *et al*., ventilation may have resulted in significantly higher personal exposure to particles originating from ambient sources [29]. Our study also showed the same results where students’ personal PM_2.5_ exposure levels were more strongly associated with ambient PM_2.5_ air pollution. It is supposed that ventilation hobby of Chinese people either in summer or winter (91.1% children family had the hobby of ventilation every day even in winter) and less residential building air tightness in China are the probably reasons for this problem. According to a report by Wang *et al*., for an apartment with normal air tightness and without any HVAC-filter system, most indoor PM_2.5_ was originated from outdoor-generated particles and closed windows can only play a very weak role on the decline of indoor PM_2.5_ concentrations[11].Several studies have demonstrated that central HVAC (heating, ventilating, and air-conditioning) in residences, and children’s gender were likely to have a significant impact on exposure to particulate matter [13, 30–33]. However, in our research, no significant impact was observed for central HVAC or gender differences to personal PM_2.5_ exposure levels. Therefore, we consider these results in terms of the limitations of the research design and perform further research if necessary. Our investigation was performed in winter of 2013, during which an extremely rare air pollution event occurred in Shanghai because of excessive emissions and unique meteorological conditions. Although students probably reduced the duration of their outdoor activities during this period, these students were still exposed to high PM_2.5_ ambient contamination. In addition, the duration of sports activities for these children was significantly insufficient both at home and school, according to our research. One probable reason was that these children preferred to stay indoors during the extremely rare air pollution event but another problem that surfaced was the heavy burden of schooling in China, which is of significant concern. This issue should be considered by government authorities.

Increasingly, scientists have reported that personal exposure to air pollutant concentrations was strongly associated with the daily activity patterns, lifestyle, and the different microenvironments in which they frequently occurred [5, 34, 35]. Based on our results from PCA regression, children’s personal PM_2.5_ exposure was associated with various factors. For example, ambient air pollution, meteorological conditions and children’s age were strongly correlated with personal PM_2.5_ exposure levels. This result demonstrated again that PM_2.5_ from ambient origins predominantly contributes to personal PM_2.5_ exposure in China[36]. Multiple linear regression result showed that children’s age was strongly correlated with personal PM_2.5_ exposure levels, which was probably because of the fact that an elder child had longer time of and more intense outdoor activities, so that their chance of exposure to outdoor PM_2.5_ is higher compared with a younger child. Nonetheless, storey height of classroom and students’ transportation mode to school was negatively correlated with students’ exposure levels. The PM_2.5_ exposure levels in students were different depending on the different storey height of classroom and modes of transportation to school. Therefore, we recommend that children should take cars, buses or the subway to school as much as possible, especially during periods of haze or serious pollution.

An important advantage of real-time continuous instrumentation is the ability to determine short-term temporal and spatial variation. Although real-time sampling has greater measurement error than the US federal reference method (FRM) gravimetric PM_2.5_ samplers, it can provide useful real-time information. As reported by Jeff D. *et al*., the 24-h average DustTrack levels are well correlated with FRM levels with a slope of 2.57 and an R^2^ of 0.859 (*P* <0.0001) [37]. In our research, the two laser diode photometer monitors were approximately 2.25- and 2.59- fold as high as the gravimetric measurements with the R^2^ of 0.799 and 0.769 (*P* <0.001), respectively. Therefore, Such data are of use especially if any biases are consistent and when statistical adjustment can be made and these types of continuous measurements could be accepted and used in many studies [38–40]. However, we had to admit that statistical power of the multiple linear regression results in our research was low because of a small sample size. Therefore, we should consider further studies with a larger sample size, while also performing the study throughout all seasons and improving our monitoring method in the future.

We made use of personal sampling and micro-environmental monitoring to explore children’s PM_2.5_ exposure levels. Meanwhile, we analyzed children’s real-time exposure variation in a day and explored the probable impact of daily exposures. The method we used can be used as a reference, especially for susceptible populations such as the elderly, pregnant women, and other groups. In contrast, children’s exposure levels reported here were monitored during an extremely polluted episode in Shanghai, which could help us understand children’s exposure to PM_2.5_ in China more generally. Our study findings should be of interest to the government or relative institutions so that appropriate policies to protect children can be considered.

## Conclusions

These primary school students in Shanghai were exposed to high concentrations of pollutants during a serious haze period in winter of 2013. All the median personal exposure and microenvironment PM_2.5_ concentrations greatly exceeded the daily 24-h PM_2.5_ Ambient Air Quality Standards of China, the USA, and the World Health Organization (WHO). The concentration of time-weighted average (*TWA*) exposure level of all the students was 137.01 μg/m^3^. Based on our research, children’s personal PM_2.5_ exposure was associated with various factors. Besides ambient air pollution and meteorological conditions, storey height of the classroom, and mode of transportation to school were all significantly correlated with personal PM_2.5_ exposure levels. Since outdoor air pollution has been an important source of PM_2.5_ exposure among children, the Chinese government must take strong environmental actions to combat haze. On the other hand, school children and families also should take measures to prevent personal exposure of pollution to children. What is equally important is that we need to pay more attention to the problem of insufficient physical activity time for primary school students, as this can result in public health issues later in life. Only through the joint efforts of governments, schools, and families can we minimize the health risks of air pollution to children.

## Supporting information

S1 Database Data of PM_2.5_ concentrations obtained from microenvironments. (SAV)

S2 Database Data of PM_2.5_ concentrations obtained from personal exposure measurements. (SAV)

S3 Database Data of ambient air pollutants concentrations and meteorological variables. (SAV)

S4 Database Data of time-activity pattern and multiple linear regression explore. (SAV)

S1 Protocol Operation protocol of the measurements. (DOC)

S1 Appendix English translated questionnaire. (DOC)
