# Supplementary material for: Personal exposure measurements of school-children to fine particulate matter (PM2.5) in winter of 2013, Shanghai, China
Source: PLoS One. 2018 Apr 2;13(4):e0193586. doi: 10.1371/journal.pone.0193586 (PMC5880346; doi:10.1371/journal.pone.0193586)
Supplement: S1 Protocol — (DOC) [file pone.0193586.s005.doc]

**S1_Protocol**

**Operation Protocol of the Measurement**

**Personal Exposure Measurements of School-children to Fine Particulate Matter (PM_2.5_) in winter of 2013, Shanghai, China**

- **School selection**

School selection should be based on air pollution and human health monitoring data in the target research area in order to be representative. In our study, two primary schools were selected based on air pollution and human health monitoring data in Shanghai and the cluster sampling principle. One school was located in the downtown area and the other was located in the suburban area. Both schools were less than 5 km away from the nearest fixed air-monitoring station of Shanghai Environmental Monitoring Centre.

- **Field selection**

Based on the stratified random principle, 3 classrooms of the 3^th^, 4^th^ and 5^th^ grade of each primary school and 1 main corridor close to the selected classrooms in each primary school were selected. The playground of each school was selected as the outdoor exposure monitoring spot.

- **Subject selection**

Following the principles of gender and grade equity and voluntary participation, 57 children of grades 3, 4 and 5 of the two primary schools were recruited. All students lived in the communities close to the two selected schools. Our study was approved by the ethics committee of Shanghai Municipal Centre for Disease Control and Prevention. Informed consent forms were signed by the parent or guardian of each participant before the study was initiated.

- **Questionnaire survey**

Information on living environment of each participant was collected through a questionnaire. The main variables included general information, residential information, living condition and lifestyle.

- **PM_2.5_ measurements inside the campus microenvironment**

During the on-campus period (from 8 a.m. to 4 p.m.), continuous PM_2.5_ measurements were conducted simultaneously in several spots inside the school campus with TSI DUSTTRAK^TM^ DRX monitoring apparatus (Model 8533, TSI Inc. Paul, MN, USA). All sampling instruments were calibrated with the manufacturer’s high-efficiency particulate filter before sampling.

Classrooms: Two samplers were placed in each classroom from 8 a.m. to 4 p.m. for two days based on the diagonal distribution principle, with one in the front of the classroom and the other in the rear. During the sampling period, all classrooms retained ventilation habits as usual, including the opening and closing of windows and doors.

Main corridors: One sampler was placed in each main corridor from 8 a.m. to 4 p.m. for 6 days

Playgrounds: One sampler was placed in the playground from 8 a.m. to 4 p.m. for 6 days.

All sampling instruments were placed approximately 1 meter away from walls and other barriers, and were at a height of 1.2 meters above ground level, which was approximately at the height of the breathing zone of children. The sampling flow rate was 3 l/min, and values were recorded continuously over 8 hours with time intervals of 1 min. The limit of detection (LOD) of the sampling instrument was 1μg/m^3^.

- **Personal measurements outside campus**

During the off-campus period (from 4 p.m. to 6 a.m. the next day), PM_2.5_ concentrations were collected through a set of real-time laser diode photometers (model no. SidePak^TM^ AM510, TSI Inc, USA), which was placed in a small bag. A sampling air inlet was fixed in the vicinity of the student’s breath zone. The sampling flow rate was 1.7 l/min with time intervals of 1 min. The limit of detection (LOD) of the sampling instrument was 1μg/m^3^.

All participants were asked to carry the sampling bags on back from 4 p.m. to 8 a.m. the next day, excluding the sleeping and showering periods. When the bag was taken off, it was required to be placed within the breathing zone of the participant. All students returned sampling bags the next morning. Each instrument was cleaned, greased and batteries were replaced before redistribution to the same student before school finishes that afternoon. All participating students were required to finish personal sampling for a continuous 2 days. All instruments were reset before sampling, according to the manufacturer’s instructions

- **Outside monitoring data collection**

Data of ambient air pollutants (e.g. PM_10_, PM_2.5_, NO_2_ and SO_2_) and meteorological indicators (e.g. temperature, humidity, and wind speed) of the fixed air-monitoring stations close to the schools were collected from Shanghai Environmental Monitoring Centre (http://www.semc.com.cn/aqi/home/Index.aspx) and Shanghai Meteorological Service, respectively.

- **Time-activity Diary**

Each student was asked to record his/her time activity patterns on a diary, which included time, activities, and location. This diary consisted of two parts, the on-campus part based on the school timetable and the off-campus part with a record interval of 30 minutes.

- **Calculation of gravimetric and real-time sampling of PM_2.5_ Concentrations**

The two kinds of direct reading instruments (Model 8533 and SidePak^TM^ AM510) and one type of gravimetric sampling instrument (Sven/Leckel LVS3) were set simultaneously in several specific micro-environments in order to find the relationship between gravimetric sampling results and real-time sampling results. The micro-environments includes:

Beside elevated roads: The instruments were placed 10 meters away from an elevated road for 4 days, 8 hours per day.

Tobacco smoking: The instruments were set in a 15-square-meter room with no air exchange for 3 days, 8 hours a day. One smoker would smoke in the room 1 or 2 times a day，for 10 minutes each time.

Mosquito coils burning: The instruments were set in a 15-square-meter room with no air exchange for 3 days, 8 hours a day. Every day mosquito coils were burned for 2 hours in the morning and afternoon, respectively.

Kitchen: The instruments were set in a kitchen with ventilation system for 3 days, 8 hours a day.

Indoor environment: The instruments were set in a 15-square-meter room with no air exchange for 8 days, 8 hours a day.

For gravimetric sampling instruments (Sven/Leckel LVS3, Germany), PM_2.5_ were collected on 47mm PTFE filters (pore size 2 μm, EmfabTM Filters TX40Hi20-WW, PALL Inc., USA) at a flow rate of 2.3 m^3^ h^-1^. All filters were pre-conditioned at a constant air temperature of 25℃±1℃ and constant relative humidity (RH) of 55%±10% for at least 24 hours and then weighed using a microbalance (CP2250, Sartorius, Germany) with 0.00001 g precision before and after sampling. All real-time sampling results were calibrated with the PM_2.5_ gravimetric mass concentrations.

- **Statistical analysis**

All data were examined for validity and complied with our standard operating procedures. Flagged data were removed when battery failure or disconnected power supply was detected. All statistical analysis was performed by SAS for Windows (version 9.4; SAS Institute Inc., 2003) and the level of significance was defined as *P* <0.05 (2-tailed)**.**
