# Supplementary material for: Personal exposure measurements of school-children to fine particulate matter (PM2.5) in winter of 2013, Shanghai, China
Source: PLoS One. 2018 Apr 2;13(4):e0193586. doi: 10.1371/journal.pone.0193586 (PMC5880346; doi:10.1371/journal.pone.0193586)
Supplement: S1 Appendix — (DOC) [file pone.0193586.s006.doc]

**S1_Appendix**

**English Translated Questionnaire**

**Personal Exposure Measurements of School-children to Fine Particulate Matter (PM_2.5_) in winter of 2013, Shanghai, China**

Questionnaire NO：_______ _

Name： Student ID： Grade： Class：

Address：

Telephone number：

**1. General Information**

1. Gender： ①Male ②Female
2. Date of birth: ____________(Month) _______ (Day) _______ (Year)
3. Education background of the father:
4. Below Primary school ② Primary school ③Junior high school

④ High school ⑤Vocational school ⑥Bachelor ⑦Master or above

1. Education background of the mother:
2. Below Primary school ② Primary school ③Junior high school

④ High school ⑤Vocational school ⑥Bachelor ⑦Master or above

1. Occupation of the father:

①Worker ②Farmer ③Soldier ④Civil servant or employee of enterprises/institutions ⑤Professional Personnel ⑥Education and training ⑦Commerce and Service personnel ⑧Foreign-funded enterprise employee ⑨Retiree ⑩Unemployed Others【please specify】_______

1. Occupation of the mother:

①Worker ②Farmer ③Soldier ④Civil servant or employee of enterprises/institutions ⑤Professional Personnel ⑥Education and training ⑦Commerce and Service personnel ⑧Foreign-funded enterprise employee ⑨Retiree ⑩Unemployed Others【please specify】_______

**2. Living Condition**

1. How many years have your child lived here? ______years
2. How many people does your child live with？_________________
3. Who does your child live with?  (multiple choice)

①Parents ②Grandparents ③ Other relatives ④Others 【please specify】 __

1. What is the building type of your residence？

①Bungalow ②Old public house ③Multi-storey residential building (small or high-rise residential buildings) ④House ⑤Simple residence ⑥Residence built by yourself ⑦Others【please specify】__________

1. Which floor does your child live on？ ________
2. How many years have your residence been built？ ______years
3. How big is your residence? Square meters
4. When was the last time that you decorated your house, including changing floors and brushing walls.
5. In less than 6 months ②In less than 1 year ③In less than two years

④For more than 2 years

1. Have you used the following material while decorating your house? (multiple choice)

①Paint ②Wood based panel ③Coating ④Wallpaper ⑤Marble ⑥Ceramic tile ⑦Wood floor ⑧Aluminum alloy products ⑨Other material, 【please specify】

1. How long did you wait before moving in after finishing decoration?
2. Less than 3 months ②3 to 6 months ③6 months to a year ④1 to 2 years ⑤More than 2 years
3. Have you or your family had any allergic reactions after moving into the decorated house?

①Yes ②No  **(Please skip to 2.13)**

1. Who have had an allergy reaction? (multiple choice)

①The child ②The child's parents ③The child's grandparents ④other relatives

1. What is the decoration material of your house floor? (multiple choice)

①Carpet ②Wood ③Plastic ④no decoration ⑤Marble ⑥Ceramic tile ⑦ Others【please specify】___

1. What is the decoration material of the walls in your residence? (multiple choice)
2. Paint ②Wood based panel ③Wall carpet ④Wall paper ⑤Ceramic tile ⑥Others 【please specify】___
3. Have you bought any new furniture in the past year?

①Yes ②No

1. Does your child have his/her own room?
2. Yes **(Please skip to 2.18)**  ②No
3. If not, how many people live in the same room with your child?
4. Is there an air conditioner in your child’s bedroom or living room?

①Yes ②No  **(Please skip to 2.20)**

1. Is the air conditioner cleaned or disinfected regularly?

①Yes, once a year ②Yes, twice a year ③Yes, three times a year ④Yes, four times a year ⑤No, hardly cleaned or disinfected

1. What kind of fuel do you use for cooking?

①Coal ②Coal gas ③Natural gas ④Electricity ⑤others 【please specify】___

1. What kind of ventilation do you use in your kitchen?

①No ventilation ②Exhaust fan ③[Range](http://dict.youdao.com/w/smoke exhaust ventilator/" \l "keyfrom=E2Ctranslation) hoods ④Others 【please specify】___

1. How often do you use ventilation while cooking?
2. Every time during cooking ②Sometimes ③Never
3. What are the heating sources used in your residence?

①Central heating ②Coal stove with chimney ③Coal stove without chimney ④Electric heater ⑤Air conditioner ⑥Coal or natural gas heater ⑦No heating

1. Does anyone smoke in your residence?

①Yes ②No

1. Does anyone living with the child smoke in your residence?
2. Yes, almost every day since the child was born.
3. Yes, occasionally since the child was born.
4. No  **(Please skip to 2.27)**
5. In which part of the residence do they often smoke? (multiple choice)

①Living room ②Bedroom ③Kitchen ④Bathroom ⑤Balcony or patio ⑥Other【please specify】 ___

1. How many days was your child exposed to secondhand smoke in the past 7 days?

①Zero ②1 day ③2 days ④3 days ⑤4 days ⑥5 days ⑦7days ⑧7 days(every day)

1. What is the distance between your residence and the nearest street or highway？__________meters.
2. Is the traffic volume of the street/highway heavy or small?
3. Very heavy ②Fairly heavy ③Small ④Very small
4. Are there any factories with smell or big tall chimneys near your residence?

①Yes, please specify the type of the factory____________________, and the distance between your residence and the factory is around___________meters ） ②No

**3. Life style**

1. Is your child picky with food?

①Yes ②No

1. What are the favorite foods of your child? (multiple choice)

①Meat (pork, beef, mutton, chicken) ②Fish and shrimp ③Vegetables ④Fruits ⑤Milk ⑥Other【please specify】

1. Have you ever raised any pets in your residence?

①Yes , for __________ years ②No  **(Please skip to 3.6)**

1. What kind of pets did you have?
2. Dogs ②Cats ③Birds ④Rabbits ⑤Fish ⑥Other【please specify】
3. Does your child play with pets often?
4. Yes ②No
5. Are there any plants in your house?

①Yes ②No

1. Do you often put flowers in your house?

①Yes ②No

1. How often do you open windows in summer and fall?
2. Every day ②Every 2 to 3 days ③Every 4 to 5 days ④Every 1 to 2 weeks ⑤1 to 2 times a month ⑥Never
3. How often do you open windows in winter and spring?
4. Every day ②Every 2 to 3 days ③Every 4 to 5 days ④Every 1 to 2 weeks ⑤1 to 2 times a month ⑥Never
5. In the past year, how often do you use the vacuum cleaner?

①Every day ②Every 2 to 3 days ③Every 4 to 5 days ④Every 1 to 2 weeks ⑤1 to 2 times a month ⑥Never

1. In the past year, how often do you sweep or mop the floor?
2. Every day ②Every 2 to 3 days ③Every 4 to 5 days ④Every 1 to 2 weeks ⑤1 to 2 times a month ⑥Never
3. How often do you change the bed sheet?
4. Every day ②Every 2 to 3 days ③Every 4 to 5 days ④Every 1 to 2 weeks ⑤1 to 2 times a month ⑥Never
5. Do you use the following household chemicals? (multiple choice)
6. Mothballs ②Air fresher ③Dehumidizer ④Perfume ⑤Other, 【please specify】 ⑥Never
7. Does your child have fluffy toys?

①Yes. but he/she doesn’t like playing with them ②Yes, he/she likes playing with them very much ③No

1. How often does your child participate in outdoor activities after school?
2. Every day ②Every 1 to 2 days ③Every 2 to 3 days ④ Every 3 to 4 days ⑤Once a week ⑥Almost never  **(Please skip to 3.17)**
3. How many hours does your child spend on outdoor activities each time? ___________hours
4. What is the main transportation of your child to school every day?
5. On foot ②By bus ③By subway ④By school bus ⑤By bike on his/her own ⑥By bike by the family members ⑦By car ⑧Other【please specify】_______
6. Person completing the questionnaire:

①Child's father ②Child's mother ③Child's grandparents ④Other【please specify】_______

Investigation Date: 2013_____Month____Date

**Thank you for your cooperation！**

The reviewer:________________

Audi date___________________
